
JOURNAL INFORMATION
==============================
Journal ID: ijcoms

IJQHC Communications

EISSN: 2634-5293

ARTICLE INFORMATION
==============================
PMCID: PMC8344417
DOI: 10.1093/ijcoms/lyab010
Article ID: lyab010
Article version: 1
Subjects: Commentary, AcademicSubjects/MED00862

State of Quality in the COVID-19 Era “Initiatives and Priorities for Improving Quality in the Future” Reflections from the EMR Region

Araj Salma Jaouni Chief Executive Officer (CEO), HealthCare Accreditation Council (HCAC), Amman, Jordan

Hassan Samar Technical Officer, Accreditation Department, HealthCare Accreditation Council (HCAC), Amman, Jordan

Corresponding Author: Salma Jaouni Araj, Chief Executive Officer (CEO), HealthCare Accreditation Council (HCAC), Amman 11492, Jordan. Phone: + 962 79 6288477; Email: sjaouni@hcac.com.jo
Electronic publication date: 2021 Jul 12
Electronic Location ID: lyab010
Received 2021 May 22; Accepted 2021 Jul 11; 2021 Jul 6
Copyright: © The Author(s) 2021. Published by Oxford University Press in association with International Society for Quality in Health Care.
Copyright year: 2021
License: This is an Open Access article distributed under the terms of the Creative Commons Attribution License (http://creativecommons.org/licenses/by/4.0/), which permits unrestricted reuse, distribution, and reproduction in any medium, provided the original work is properly cited.
License URL: https://creativecommons.org/licenses/by/4.0/

edited-state: accepted-manuscript
article-lifecycle: PAP

==============================
### State of Quality in the COVID-19 Era

### "Initiatives and Priorities for Improving Quality in the Future"

### ***Reflections from the EMR Region***

Salma Jaouni Araj[1], Samar Hassan[2]

[1] Chief Executive Officer (CEO), HealthCare Accreditation Council (HCAC), Amman, Jordan

[2] Technical Officer, Accreditation Department, HealthCare Accreditation Council (HCAC), Amman, Jordan

***Corresponding Author**:

Salma Jaouni Araj

Chief Executive Officer (CEO), HCAC

PO Box 11492, Amman, Jordan

Phone: + 962 79 6288477

Email: sjaouni@hcac.com.jo

ACCEPTED MANUSCRIPT

**Running title:** State of Quality in the COVID-19 Era- Reflections from the EMR Region.

**State of Quality in the COVID-19 Era**

**Initiatives and Priorities for Improving Quality in the Future**

***Reflections from the EMR Region***

© The Author(s) 2021. Published by Oxford University Press in association with International Society for Quality in Health Care.

Compromising 9% of the worlds population (6), nearly two thirds of the Eastern Mediterranean Region (EMR)'s countries are directly or indirectly affected by conflict, natural disasters, and political instabilities.(1) Others are wealthy countries classified under upper income by the World Bank and definitely we have no regional leadership, cooperation or clarity in data collection or reporting. All have been affected by COVID-19 and have worked to improve quality of care.

**Box 1. Humanitarian Overview of the EMR Region:** (31).

Ramifications of the pandemic were exacerbated by existing humanitarian crisis in many countries with armed conflict (32)

- Eight EMR countries affected by conflict and humanitarian emergencies—Afghanistan, Iraq, Libya, the occupied Palestinian territory, Somalia, Sudan, Syria, and Yemen.
- Over 12 million people are internally displaced across the region.
- Escalating hostilities in parts of Syria, Yemen and Libya continue to force people to flee their homes and exacerbate civilian suffering,

Looking at information that is available from governments and the WHO EMR office, we offer some insights on how well prepared our healthcare systems have been to navigate through this turbulent environment, what are the lessons learned so far, and how are we moving forward.

As of 11 March 2021, the 22 countries of the EMR have reported a total of 6,741,645 cases and 148,733 deaths (2). These numbers of course, are missing the real numbers of death in countries where registration and follow up is weak. The Region deals with these cases, while grappling with existing challenges prior to the pandemic including serious shortages of health workforce, shortages of essential medicines, in most low-middle income countries, inadequate service delivery for assistive technology in many countries, and extremely limited access to medical devices in low- and middle-income countries and even some high-income countries. (1) Despite that, most countries in the EMR region established high-level national multisectoral coordination mechanisms and developed national plans covering the nine pillars of the WHO COVID-19 Strategic Preparedness and Response Plan.(3)

**Box 2. Nine pillars of the WHO COVID-19 Strategic Preparedness and Response Plan:** (10)

- Country-level coordination, planning, and monitoring
- Risk communication and community engagement
- Surveillance, rapid response teams, and case investigation
- Points of entry, international travel, and transport
- National laboratories
- Infection prevention and control
- Case management
- Operational support and logistics Maintaining essential health services and systems

While the pandemic has presented an array of challenges from managing the supply chain for personal protective equipment (PPE), to adjusting workforce capacity, to coping with financial loss(4), it has also driven many health systems across the region to embark on new models of health service delivery, highlighted the need for taking unorthodox approaches, and triggered a sustained demand for innovation (11). Unprecedented strides have been taken in the EMR region, in the areas of [1] digital & telehealth expansion, [2] emergency care system strengthening, and [3] research and knowledge sharing – possibly a silver lining!

In the area of **Telehealth**, a rapid expansion has been seen for payment systems and regulation, while allowing for the provision of remote access to quality healthcare services without increasing the risk of transmitting infection(5), and with an attempt to address the health care needs of patients with chronic

ACCEPTED MANUSCRIPT

diseases, and mental health conditions. This has been especially beneficial for the EMR region, where there is a double burden of communicable and noncommunicable diseases;the region has some of the highest NCD prevalence rates globally and future projections indicate an alarming increase. The COVID-19 pandemic lockdowns affected people's access to medications for management of their noncommunicable diseases (NCDs) and EMR countries but adaptations were made as illustrated in the below table:

*Table 1: Telehealth initiatives-NCDs Services in the EMR region* (6)

| Country | **Initiatives** | Details |
|---|---|---|
| Jordan | **The use of a web-based tool to improve access to NCD medications** | • An electronic platform called "e-Med Hakeem" was established to meet the prescription refill needs. For the insured patient to receive his or her prescription refill.<br>• The e-Med platform aims towards enabling easy access of patients to their monthly recurring medications through a simple and user-friendly interface |
| Oman | **Phone Consultation & Counseling** | • Phone consultations providing instructions on how to self-manage and share blood pressure measurements, blood sugar levels, etc.<br>• Sending reminders for appointments, cancellations and rescheduling.<br>• Sending reminders for medication prescriptions and collection times. |
| Qatar | **Virtual video consultations.**<br>**Home delivery of medications.** | • This service provides remote access to health care services.<br>• Home delivery of medications provides medication fulfillment and refills through home delivery to reduce footprint. |
| | **Self-assessment chatbot.** | • This is a computer program that anyone can talk to and is being used to facilitate triaging. It is available in six languages: Arabic, English, Hindi, Malayalam, Tagalog and Urdu. |
| Sudan | **Telephone Hotlines** | • A telephone hotline accessible to people living with NCDs in all states of Sudan. The hotline offers many services, including the provision of medical consultations, triaging, medication prescriptions, health education, counselling, and referrals.<br>• WhatsApp API complements the hotline to facilitate the health education, counselling, referrals, and the exchange of prescriptions and lab investigations. |
| Saudi Arabia | **Telephone Service center.** | • This center provides 24/7 remote consultations and support through phone calls, and most recently, through WhatsApp as well |
| | **E-Laboratory Reporting** | • electronic laboratory investigations which enable health care providers to request the needed tests from their patients electronically who then reshare the results once done thus keeping contact at a minimum |
| | **Wasfaty app** | • The App links users to a wide network of pharmacies enabling them to fill prescriptions or refill regular medications |
| | **Mawid app** | • The App is centered around e-booking services. It helps users to book, reschedule and cancel appointments at primary health care centers, as well as for referral services |
| Egypt | **Chefaa Health-Tech** | • Manages chronic patient's monthly prescriptions-with the use of AI and GPS technology.<br>• 24/7 chat support is managed by licensed pharmacists who help assure and serve patients, answering their questions in light of the published instructions by the Egyptian Ministry of Health (MOH).(7) |
| | **Vezeeta Health-Tech** | • Connects users with 21,000 locally licensed doctors across 41 medical specialties.<br>• Launched a free medical consultation service for anyone experiencing flu-like symptoms. (7) |
| UAE | **E-Visits**<br>**Social Media** | • This service provides people living with NCDs with phone or video consultations, in addition to e-prescriptions and home delivery of medications.<br>• Social media is being used to provide people living with NCDs with information and messaging around COVID-19, their susceptibility to more severe symptoms due to their conditions, and the importance of avoiding unhealthy behaviors like tobacco use, unhealthy diets, etc. |

ACCEPTED MANUSCRIPT

Mental Health Services were also addressed through Telehealth Initiatives with an attempt to ease the stress and pressure which was sparked by the pandemic and its measures. In response, EMR countries established interventions to meet the growing demand for mental health and psychosocial support services. Some examples of EMR initiatives are presented in the table below:

*Table 2: Telehealth initiatives-Mental Health Services in the EMR region*(6)

| Country | Initiatives | Details |
|---|---|---|
| Jordan | Mental health hotlines | • The hotlines are manned by specialist clinical psychologists who provide mental health and psychosocial support services to callers.<br>• Providing medication fulfillment and refills through home delivery by volunteer doctors. |
| Oman | phone consultations | • Counseling and support.<br>• Instructions on self-management practices.<br>• Reminders for appointments, cancellations and rescheduling.<br>• Reminders for Medication prescriptions and collection times. |
| Qatar | Mental health helpline | • The helpline is a tele-psychiatry line that staffs a team of mental health professionals who assess and provide support to callers. Callers are categorized into four main groups: children and parents, adults, older people, and frontline healthcare workers. The helpline operates on three intervention levels.<br>• First intervention level is triaging to assess people's needs.<br>• Second intervention level is provision of psychological support.<br>• Third intervention level is referral to specialized psychiatric services |

Another area with an expansion of initiatives was in **Emergency Care Systems Strengthening,** where many countries aimed to have more agile and resilient response systems. Several models were implemented in the EMR to ensure effective response including system activation and field facility communication strategies. In 2018, only 13 of 22 EMR countries reported having a national emergency care access number required for system activation (8). However, in response to the WHO's key recommendation to strengthen the health system response to COVID-19, as 20 of 22 EMR member-states and Palestine have established ad-hoc national or regional COVID-19 hotlines for the public to access information, report suspected cases, and be screened for symptoms of COVID-19.(9)

Another key functional component of emergency care system is field-to-facility communication. Such pre-arrival notification and coordination between ambulance services is rare in the EMR region (10). As part of COVID-19 response, many countries established pre-arrival notification protocols, in order to ensure coordination to allow for an adequate clinical response to patients with COVID-19 respiratory problems. Additionally, the pre-arrival protocols ensure that emergency care units are prepared for critical and time-sensitive cases (e.g. Acute myocardial infarction, stroke, trauma etc.).(11)

Among the weaker areas in healthcare decision and planning is the lack of data and research to guide interventions. However, the COVID-19 pandemic has stimulated an enormous amount of research by scientists across various disciplines and in the EMR who have contributed to the research initiatives and the collaborative knowledge sharing, within the following domains:

- **Treatment Trials**: 12 countries in the EM Region have enrolled in the **solidarity clinical** trial which was launched by WHO and partners to help find an effective treatment for COVID-19 (12)

ACCEPTED MANUSCRIPT

- **Collaborative Evidence-based research**: Several countries of the EMR participated in research studies that enable better understanding of the disease epidemiology, and cover key areas of research and innovation (e.g. digital health, as well as outbreak investigation and clinical management) as identified by WHO as key research areas for COVID-19 (13)
- **Healthcare Providers knowledge, Attitude, & Practices** were intensively examined across the region, as many studies were published from countries including the Gulf, Levant, and Africa highlighting the main challenges faced by healthcare providers and the main actions needed to alleviate the heavy burden they face during the pandemic(14)(15)(16)(17)(18)(19)(20)(21)(22)(23)(24)(25)(24)(26)(27)(28)
- **COVID-19 Vaccine Clinical Trials:** The EMR region has played an active role in the approval process of one of the earlier vaccines to be developed and approved (Sinopharm vaccine). The vaccine trial was a partnership between Sinopharm's China National Biotec Group (CNBG) and Abu Dhabi-based artificial intelligence and cloud computing company Group 42 (G42). It was widely tested in the UAE, Egypt, Bahrain, and Jordan. The vaccine was then approved and rolled out in many EMR countries beginning January 2021 (29)

***Implications for the Future***

While still facing the burdens of the pandemic, countries in EMR are forced to reassess and think creatively about healthcare quality in the post-COVID-19 world. In order to become resilient, countries in the EMR must continue efforts to build strong systems for health security, ensure sustained investments, and insist on strong governance of preparedness for future emergencies.(30). Though many interventions were catalyzed by panic and reactive modes, it is imperative to sustain the learning and build on the investments to date, such as the use of telehealth, community outreach, supporting research for decision making, strengthening emergency response, and developing more people-centered services though call centers, primary healthcare teams, community involvement and designing interventions that speak to citizen perceptions and culture. In this way, we can maximize resources and support each other.

ACCEPTED MANUSCRIPT

### References

1. WHO Regional Office for the Eastern, Mediterranean. Health and well-being profile of the Eastern Mediterranean Region: an overview of the health situation in the Region and its countries in 2019 [Internet]. Cairo; 2020 [cited 2021 Mar 11]. Available from: https://applications.emro.who.int/docs/9789290223399-eng.pdf?ua=1

2. WHO EMRO | Corona virus | COVID-19 | Health topics [Internet]. [cited 2021 Mar 11]. Available from: http://www.emro.who.int/health-topics/corona-virus/index.html

3. Operational planning guidance to support country preparedness and response [Internet]. [cited 2021 Mar 12]. Available from: https://www.who.int/publications/i/item/draft-operational-planning-guidance-for-un-country-teams

4. Health Care Management During Covid-19: Insights from Complexity Science | Catalyst non-issue content [Internet]. [cited 2021 Mar 12]. Available from: https://catalyst.nejm.org/doi/full/10.1056/CAT.20.0541

5. Using Telehealth to Expand Access to Essential Health Services during the COVID-19 Pandemic | CDC [Internet]. [cited 2021 Mar 12]. Available from: https://www.cdc.gov/coronavirus/2019-ncov/hcp/telehealth.html

6. WHO EMRO | Digital innovation stories during COVID-19 | Publications | NCDs [Internet]. [cited 2021 Mar 12]. Available from: http://www.emro.who.int/noncommunicable-diseases/publications/success-stories.html?format=html

7. Egypt is accelerating digitisation in healthcare - International Finance [Internet]. [cited 2021 Mar 15]. Available from: https://internationalfinance.com/egypt-is-accelerating-digitisation-in-healthcare/

8. World Health Organization. GLOBAL STATUS REPORT ON ROAD SAFETY 2018 SUMMARY [Internet]. 2018 [cited 2021 Mar 13]. Available from: http://apps.who.int/bookorders.

9. World Health Organization. Regional Office for the Eastern Mediterranean. COVID-19 pandemic response in the Eastern Mediterranean Region, progress report of the incident management support team [Internet]. 2020 [cited 2021 Mar 13]. Available from: https://apps.who.int/iris/handle/10665/334319

10. World Health Organization Regional Office for the Eastern Mediterranean (WHO/EMRO). Emergency Care as an Essential Component of Universal Health Coverage in the Eastern Mediterranean Region. 2016.

11. Coen D, Paolillo C, Cavazza M, Cervellin G, Bellone A, Perlini S, et al. Changing Emergency Department and hospital organization in response to a changing epidemic. Emerg Care J [Internet]. 2020 Mar 17 [cited 2021 Mar 13];16(1). Available from: https://pagepressjournals.org/index.php/ecj/article/view/8969

12. Al-Mandhari A, Kodama C, Abubakar A, Brennan R. Editorial Solidarity in response to COVID-19 outbreak in the Eastern Mediterranean Region. Mediterr Heal J [Internet]. 2020 [cited 2021 Mar

ACCEPTED MANUSCRIPT

13];26(5). Available from: https://doi.org/10.26719/2020.26.5.492

13. Strengthening the collective response and accelerating readiness in the Eastern Mediterranean Region. 2020.

14. Bazaid AS, Aldarhami A, Binsaleh NK, Sherwani S, Althomali OW. Knowledge and practice of personal protective measures during the COVID-19 pandemic: A cross-sectional study in Saudi Arabia. Kumar R, editor. PLoS One [Internet]. 2020 Dec 11 [cited 2021 Mar 7];15(12):e0243695. Available from: https://dx.plos.org/10.1371/journal.pone.0243695

15. Alsahafi AJ, Cheng AC. Knowledge, attitudes and behaviours of healthcare workers in the kingdom of Saudi Arabia to MERS coronavirus and other emerging infectious diseases. Int J Environ Res Public Health [Internet]. 2016 Dec 6 [cited 2021 Mar 7];13(12). Available from: https://pubmed.ncbi.nlm.nih.gov/27929452/

16. Alshammari E, Alsaleh N, Alshammari A, Alsharif A, Alsaleh A, Alhamed M, et al. COVID-19 AWARENESS IN KUWAITI HOSPITALS. Mil Med Sci Lett. 2020;2020(3).

17. Hassan ZM. Improving knowledge and compliance with infection control Standard Precautions among undergraduate nursing students in Jordan. Am J Infect Control [Internet]. 2018 Mar 1 [cited 2021 Mar 7];46(3):297–302. Available from: /pmc/articles/PMC7115283/

18. Al Demour S, Ababneh MA, Al-Taher RN, Alrabadi AF, Jaradat AF, Abushamma FA, et al. Knowledge, Practice, and Attitude Toward COVID-19 Among Physicians in Jordan and Palestine: Cross-Sectional Study. Int J Gen Med [Internet]. 2021 Jan 13 [cited 2021 Mar 15];Volume 14:77–87. Available from: https://www.dovepress.com/knowledge-practice-and-attitude-toward-covid-19-among-physicians-in-jo-peer-reviewed-article-IJGM

19. Alser O, Alghoul H, Alkhateeb Z, Hamdan A, Albarqouni L, Saini K. Healthcare workers preparedness for COVID-19 pandemic in the occupied Palestinian territory: a cross-sectional survey [Internet]. medRxiv. medRxiv; 2020 [cited 2021 Mar 7]. p. 2020.05.09.20096099. Available from: https://doi.org/10.1101/2020.05.09.20096099

20. COVID-19 Knowledge, Attitudes and Practices (KAP) Survey: Northwest Syria - October 2020 (Round 5) - Syrian Arab Republic | ReliefWeb [Internet]. [cited 2021 Mar 15]. Available from: https://reliefweb.int/report/syrian-arab-republic/covid-19-knowledge-attitudes-and-practices-kap-survey-northwest-syria-5

21. Saadeh D, Sacre H, Hallit S, Farah R, Salameh P. Knowledge, attitudes, and practices toward the coronavirus disease 2019 (COVID-19) among nurses in Lebanon. Perspect Psychiatr Care [Internet]. 2020 Nov [cited 2021 Mar 15];ppc.12676. Available from: https://onlinelibrary.wiley.com/doi/10.1111/ppc.12676

22. Hussein N, Naqid I, Jacksi K, Abdi B. Assessment of knowledge, attitudes, and practices toward COVID-19 virus among university students in Kurdistan region, Iraq: Online cross-sectional study. J Fam Med Prim Care [Internet]. 2020 [cited 2021 Mar 15];9(9):4809. Available from: /pmc/articles/PMC7652109/

23. Abdel Wahed WY, Hefzy EM, Ahmed MI, Hamed NS. Assessment of Knowledge, Attitudes, and Perception of Health Care Workers Regarding COVID-19, A Cross-Sectional Study from Egypt. J Community Health [Internet]. 2020 Dec 7 [cited 2021 Mar 15];45(6):1242–51. Available from:

ACCEPTED MANUSCRIPT

http://link.springer.com/10.1007/s10900-020-00882-0

24. Abdelaziz A Ben, Benzarti S, Nouira S, Mlouki I, Achouri MY, Abdelaziz I Ben, et al. Attitudes of health professionals towards the response to the covid-19 pandemic in maghreb. Tunisie Medicale. 2020;98(5):324–33.

25. Fakhri N, Jallal M, Belabbes S, Khudur K, Kaddar R, Oubaasri A, et al. COVID-19 and Moroccan nursing students: A multicentre cross-sectional survey on their related knowledge, attitudes and practices. Nurs Open [Internet]. 2021 Mar 6 [cited 2021 Mar 15];nop2.790. Available from: https://onlinelibrary.wiley.com/doi/10.1002/nop2.790

26. Abdollahi M, Ghahramanian A, Shahbazi S, Rezaei F, Naghili B, Asghari-Jafarabadi M. Developing a questionnaire to assess iranian nurses' knowledge of and attitude to middle east respiratory syndrome. East Mediterr Heal J [Internet]. 2020 [cited 2021 Mar 13];26(5):506–16. Available from: https://pubmed.ncbi.nlm.nih.gov/32538443/

27. Ladiwala ZFR, Dhillon RA, Zahid I, Irfan O, Khan MS, Awan S, et al. Knowledge, attitude and perception of Pakistanis towards COVID-19; a large cross-sectional survey. BMC Public Health [Internet]. 2021 Dec 1 [cited 2021 Mar 15];21(1):21. Available from: https://bmcpublichealth.biomedcentral.com/articles/10.1186/s12889-020-10083-y

28. Health Worker's Perception Survey on COVID19: Knowledge, attitude and practice assessment in eight provinces of Afghanistan - Afghanistan | ReliefWeb [Internet]. [cited 2021 Mar 15]. Available from: https://reliefweb.int/report/afghanistan/health-worker-s-perception-survey-covid19-knowledge-attitude-and-practice

29. Dubai to roll out China's Sinopharm COVID-19 vaccine on Sunday [Internet]. [cited 2021 Mar 13]. Available from: https://www.arabnews.com/node/1800936/middle-east

30. Annual report on global preparedness for health emergencies Global Preparedness Monitoring Board A WORLD AT RISK [Internet]. 2019 [cited 2021 Mar 13]. Available from: http://apps.who.int/iris

31. Global Humanitarian Overview 2021 | OCHA [Internet]. [cited 2021 Mar 15]. Available from: https://www.unocha.org/global-humanitarian-overview-2021

32. Pettersson T, Högbladh S, Öberg M. Organized violence, 1989–2018 and peace agreements. J Peace Res [Internet]. 2019 Jul 3 [cited 2021 Mar 11];56(4):589–603. Available from: http://journals.sagepub.com/doi/10.1177/0022343319856046

ACCEPTED MANUSCRIPT
